# Is there a renoprotective value to leukodepletion during heart valve surgery? A randomized controlled trial (ROLO)

**DOI:** 10.1186/s13019-021-01402-4

**Published:** 2021-03-26

**Authors:** Espeed Khoshbin, Sally Spencer, Laurence Solomon, Augustine Tang, Stephen Clark, Elizabeth Stokes, Sarah Wordsworth, Lucy Dabner, Julia Edwards, Barnaby Reeves, Chris Rogers

**Affiliations:** 1 Department of Cardiothoracic Surgery, Freeman Hospital, High Heaton, Newcastle upon Tyne, NE7 7DN UK; 2 School of Health and Medicine, Lancaster University, Bailing, Upper Market Street, Lancaster, Lancashire LA1 4YW UK; 3 Renal Unit, Lancashire Teaching Hospitals NHS Foundation Trust, Sharoe Green Lane, Fulwood, Preston, Lancashire PR2 9HT UK; 4 Department of Cardiothoracic Surgery, Blackpool Victoria Hospital, Blackpool, FY3 8NR UK; 5 Department of Public Health, University of Oxford, Rosemary Rue Building, Old Road Campus, Headington, Oxford, OX3 7LF UK; 6 Clinical Trials and Evaluation Unit, Bristol Medical School, University of Bristol, Bristol, BS2 8HW UK

**Keywords:** Cardiac surgery, Heart valve, Cardiopulmonary bypass, Acute kidney injury, Leukodepletion

## Abstract

**Background:**

Acute Kidney Injury (AKI) adversely affects outcomes after cardiac surgery. A major mediator of AKI is the activation of leukocytes through exposure to the cardiopulmonary bypass circuit. We evaluate the use of leukodepletion filters throughout bypass to protect against post-operative AKI by removing activated leukocytes during cardiac surgery.

**Methods:**

This is a single-centre, double-blind, randomized controlled trial comparing the use of leukodepletion versus a standard arterial filter throughout bypass. Elective adult patients undergoing heart valve surgery with or without concomitant procedures were investigated. The primary clinical outcome measured was the development of AKI according to the KDIGO criteria. Secondary measures included biomarkers of renal tubular damage (urinary Retinol Binding Protein and Kidney Injury Molecule-1), glomerular kidney injury (urinary Micro Albumin and serum Cystatin C) and urinary Neutrophil Gelatinase Associated Lipocalin, as well as the length of hospital stay and quality of life measures through EQ-5D-5L questionnaires.

**Results:**

The ROLO trial randomized 64 participants with a rate of recruitment higher than anticipated (57% achieved, 40% anticipated). The incidence of AKI was greater in the leukodepletion filter group (44% versus 23%, risk difference 21, 95% CI − 2 to 44%). This clinical finding was supported by biomarker levels especially by a tendency toward glomerular insult at 48 h, demonstrated by a raised serum Cystatin C (mean difference 0.11, 95% CI 0.00 to 0.23, *p* = 0.068) in the leukodepleted group. There was however no clear association between the incidence or severity of AKI and length of hospital stay. On average, health related quality of life returned to pre-operative levels in both groups within 3 months of surgery.

**Conclusions:**

Leukocyte depletion during cardiopulmonary bypass does not significantly reduce the incidence of AKI after valvular heart surgery. Other methods to ameliorate renal dysfunction after cardiac surgery need to be investigated.

**Trial registration:**

The trial was registered by the International Standard Randomized Controlled Trial Number Registry ISRCTN42121335. Registered on the 18 February 2014. The trial was run by the Bristol Clinical Trials and Evaluation Unit. This trial was financially supported by the National Institute of Health Research (Research for Patient Benefit), award ID: PB-PG-0711-25,090.

**Supplementary Information:**

The online version contains supplementary material available at 10.1186/s13019-021-01402-4.

## Introduction

Following cardiac surgery the overall incidence of acute kidney injury (AKI) as defined by RIFLE (Risk, Injury, Failure, Loss of kidney function) criteria is 23% [1, 2]. Patients undergoing valvular heart surgery are at a particularly higher risk of AKI [3]. Heart valve surgery is an independent risk factor for the development of AKI which has been detected in up to 40% of these patients [4, 5]. This may be due to a relatively longer period of cardiopulmonary bypass.

AKI is associated with a twofold increase in mortality, prolonged ICU stay, chronic ill-health and a 1.6 fold increase in the financial cost of care with the risk to costs ratio escalating with the severity of AKI [6, 7]. Therefore AKI significantly and adversely affects prognosis and health outcome after cardiac surgery [8, 9].

Central to the complex etiology of peri-operative renal damage is the systemic inflammatory response to cardiopulmonary bypass (CPB). The important role of leukocyte activation in CPB-associated inflammation is universally recognized [10–13]. It has been previously hypothesized that the inherent nephrotoxic effect of CPB may be significantly reduced by incorporating a leukodepletion filter into the circuit (LG-6 LeukoGuard® Leukocyte Reduction- Pall Corporation) to physically remove activated leukocytes [14–17]. However the effectiveness of leukodepletion in reducing CPB-associated renal impairment has not been fully evaluated [18].

We tested the hypothesis that leukocyte depletion during bypass would ameliorate renal injury in patients undergoing heart valve surgery as this group of patients have a higher risk of developing bypass related AKI [4]. Previous study of a low risk cohort undergoing coronary surgery alone did not demonstrate clinical benefit [15].

## Methods

AKI was defined by Kidney Disease Improving Global Outcomes (KDIGO) criteria (Table 1) in patients undergoing heart valve surgery.

**Table 1:** Kidney Disease Improving Global Outcomes (KDIGO): primary outcome defined by this criterion for acute kidney injury

Stage	Serum creatinine (SCr) criteria	Urine output criteria
1	Increase ≥26 μmol/L within 48 h or increase ≥1.5 to 1.9 x reference baseline SCr	< 0.5 mL/kg/hr. for > 6 consecutive hrs
2	Increase ≥2 to 2.9 x reference baseline SCr	< 0.5 mL/kg/hr. for > 12 h
3	Increase ≥3 x reference baseline SCr or increase ≥354 μmol/L or commenced on renal replacement therapy irrespective of stage	< 0.3 mL/kg/hr. for > 24 h or anuria for 12 h

This single-centre, double-blind study was designed as an external feasibility trial comparing two parallel groups. Patients were randomized to receive either a leukodepletion filter or a standard arterial filter as part of the bypass circuit. The filter was used throughout the bypass run from start to finish.

The frequency of AKI, clinical and sub clinical data, health-related quality of life (HRQoL) and resource use data was investigated and collected. We assessed the sub clinical effects of leukodepletion through measurement of urinary and serum biomarkers of tubular and glomerular kidney injury. Acute injury to the renal tubules was assessed by excretion of retinol binding protein (RBP) in the urine (ELISA methods). This biomarker has previously been validated in cardiac surgical patients [19, 20]. More recent evidence suggests urinary Kidney Injury Molecule-1 (KIM-1) as a superior biomarker (ELISA method) than RBP [20, 21]. Acute injury to the renal glomeruli was assessed by urinary micro Albumin (Alb) through turbimetric assay [22] and by serum Cystatin-C concentration, with potentially better sensitivity compared to glomerular filtration rate [23]. These biomarkers allow for early detection of renal injury before conventional clinical parameters such as blood urea and serum Creatinine, become abnormal.

Fresh urine samples were collected within strict time ‘windows’ for each biomarker. Urinary RBP, KIM-1 and Alb were indexed to urinary excretion of Creatinine (RBP:Cr, Alb:Cr, KIM-1:Cr) to adjust for variations in the glomerular filtration rate. Urinary Neutrophil Gelatinase Associated Lipocalin (NGAL) [24] another biomarker used in cardiac surgery to assess associated AKI and serum Cystatin C was measured as actual concentrations.

Comparative analyses of levels of biomarkers over time was carried out using mixed regression models, including main effects of group, time and the interaction of group by time. This allowed for a more precise assessment of AKI than can be achieved using the KDIGO criteria, which does not differentiate between pre-renal failure (due to volume depletion) and renal damage.

HRQoL was measured using the Minnesota Living with Heart Failure (MLHF) [25] and EQ-5D-5L questionnaires enabled the evaluation of both impact on quality of life and cost (resource use data) in a single measure [26, 27] up to 3 months following surgery.

The sample size was set at 108 participants (54 per group) who would allow the incidence of AKI to be estimated with the following precision (95% confidence interval): 31 to 50% for an incidence of 40 and 5% to 17% for an incidence of 10% (Proportions as high as 40%, and as low as 10%, have been reported to have developed AKI following heart valve surgery). This sample size also had 95% power to detect a 1.5 fold reduction in RBP:Cr for the leukodepletion filter group at the 1% level of statistical significance [15], allowing for 10% dropout. The 1.5 fold reduction is lower than that observed in a previous study for both RBP:Cr and Alb:Cr [15], but was considered clinically relevant and appropriate as the previous study may have been at risk of bias.

Inclusion and exclusion criteria are illustrated in Table 2.

**Table 2:** Inclusion and exclusion criteria

**Inclusion criteria**	
Adults aged 18–89 years having single or multiple heart valve repair or replacement as a first time or redo operation as an elective or urgent procedure (i.e. non-emergency procedure), who are able to give informed consent.	
Patients with or without concomitant procedures. Concomitant procedures may include but are not restricted to: coronary artery bypass graft (CABG), ascending aortic and/or root replacement, and ablation for atrial fibrillation.	
Baseline urea and creatinine levels are within normal range, defined as follows: urea 2.5 to 7.8 mmol/L; creatinine 45 to 90 μmol/L for women and 60 to 110 μmol/L for men.	
**Exclusion criteria**	
Baseline eGFR< 30 mls/min/1.73 m2	
Patients on renal replacement therapy	
Planned deep hypothermic circulatory arrest with cardiopulmonary bypass switched off	

Eligible patients were randomized to either the leukodepletion filter (LG-6) or the standard filter group by the perfusionist using a secure password-protected web-based interface. The process took place immediately prior to setting up the appropriate filter in order to minimize dropout between randomization and surgery. Randomization was stratified by pre-operative risk of AKI, estimated using the Cleveland CSA-AKI Severity Score [16] (four strata, score < 3, 3 to 5, 6 to 8, > 8). Computer generated random allocations were blocked using various block sizes. The sequence was prepared in advance by a statistician independent of the study team.

Participants, surgeons and research nurses were all blinded to the treatment allocation, only the perfusionist was aware of the filter used [28, 29].

Standard adult extracorporeal tubing set was used as per routine practice in conjunction with a D903 Avant membrane oxygenator (Sorin Biomedica, Gloucester, UK). In the control group, a standard 40-μm arterial line filter was incorporated into the extracorporeal circuit downstream from the pump. This was substituted with an LG6 leukocyte-depleting filter in the study group. The circuit was primed with 1500–2000 mL Plasmalyte 148 solution, 1 g magnesium sulphate and 5000 IU sodium heparin. An S3 Revolution centrifugal pump (Stöckert Instrumente GmbH, Munich, Germany) delivered non-pulsatile flow maintained at 2.4 L/min/m^2^. A target mean perfusion pressure of 65 mmHg was maintained with appropriate use of vasoactive agents. With an activated clotting time of more than 480 s, CPB was established with mild hypothermia (lowest core temperature, 32 °C) and alpha-stat management of acid-base status during cooling and re-warming. The filter wacovered and positioned below eye level so that it was not visible to the surgeon so to avoid un-blinding.

Adverse events (AE) were defined as any unfavorable and unintended occurrence including an abnormal laboratory finding, symptom or disease associated with the intervention or procedure, regardless of whether it is considered related to the intervention or procedure that occurs during the course of the study. Any untoward medical occurrence that resulted in death, was life threatening, required prolongation of hospitalization, resulted in persistent or significant disability/incapacity was recorded as a serious adverse event.

Analyses were based on a pre-specified statistical plan and performed on an intention-to-treat (ITT) basis. Continuous data were summarized using mean and standard deviation (or median and interquartile range if distributions were skewed) and categorical data as numbers and percentages. Continuous longitudinal outcomes were compared using linear mixed effects models. Model fit was assessed via standard methods and if inadequate then transformations or alternative analysis methods were sought. All analyses used the standard filter group as the reference group and adjusted for Cleveland AKI Severity Score (stratification factor). Outcomes were reported as proportions or effect sizes as appropriate with 95% confidence intervals (CI) and likelihood ratio tests were used to determine statistical significance. Biomarker concentrations below the limit of detection were imputed using the lowest detectable concentration. All analyses were performed in Stata version 14.0 (StataCorp LP, College Station, Tex) or SAS version 9.3 (SAS Institute Inc., Cary, NC). The trial was reported according to the CONSORT reporting guidelines.

## Results

**Fig. 1:**
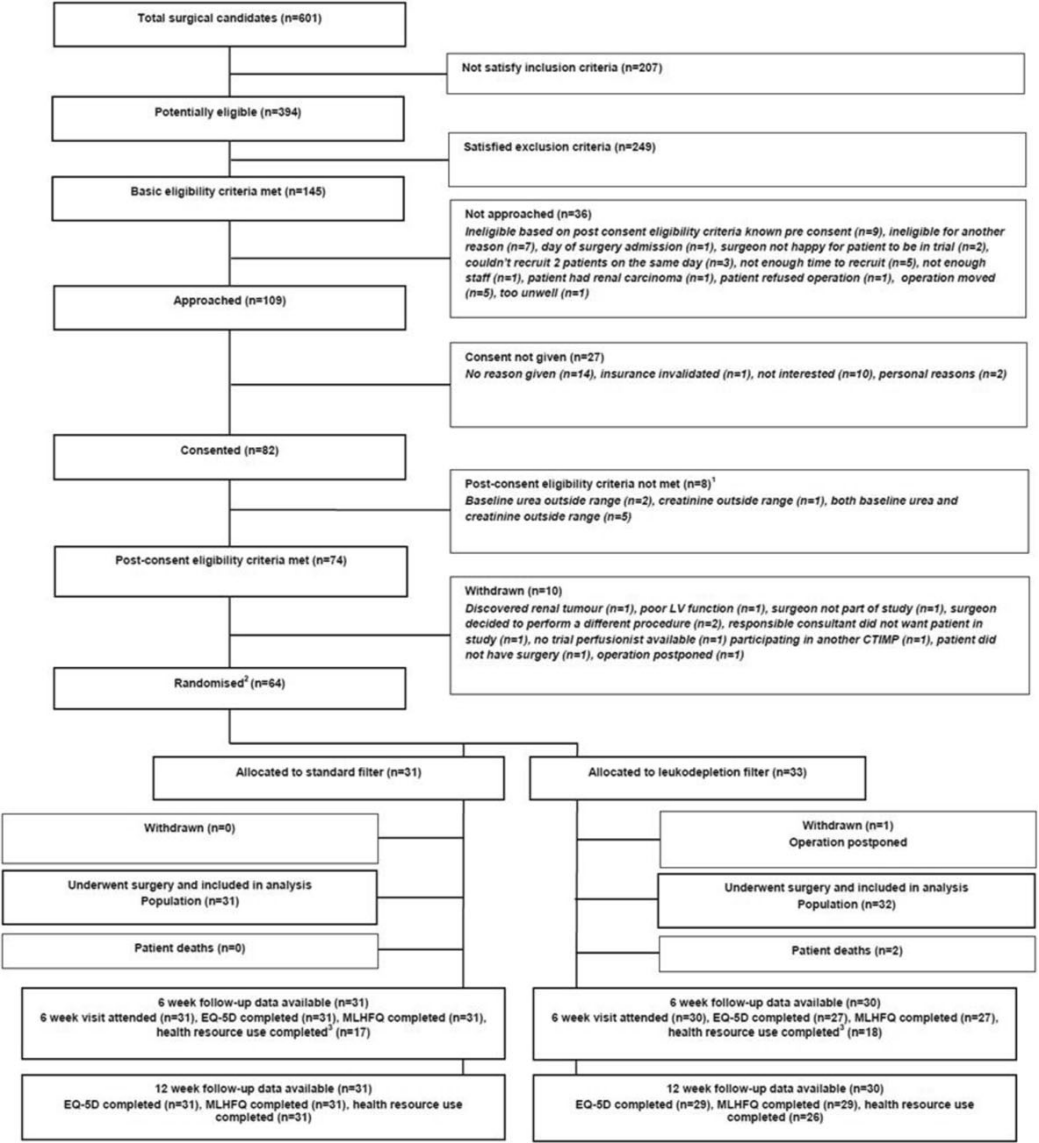
Flow of participants. *Notes* ^*1*^*: An additional 9th patient was found ineligible based on post-consent criteria but was included in the study based on repeat bloods taken* ^*2*^*: An additional 65th patient was randomized in error. As this patient did not consent and no further data was collected, this patient has not been included here.*^*3*^*: 6 week health resource use questionnaire brought in after trial started*

Three hundred ninety-four patients were potentially eligible for the trial. one hundred forty-five patients were screened and met all the basic eligibility criteria (37, 95% CI 32 to 42%). Eighty-two agreed to take part in the trial (57, 95% CI 48 to 65%). Eight patients were found to be ineligible after consent and 10 were withdrawn. Reasons for withdrawal at this stage included surgeon preference (*n* = 4), clinical condition of the patient (*n* = 2) and logistical reasons (*n* = 5), leaving 63 randomized participants, 31 to standard perfusion and 33 to receive a leukodepletion filters (Fig. 1).

There was one treatment crossover from the leukodepletion filter to the standard filter, as the pump flow was considered too high for the leukodepletion LG-6 filter. This was the only case where un-blinding occurred. One patient did not receive either intervention as the operation was abandoned due to an intra operative finding of a porcelain aorta.

Patient demographics are illustrated in Table 3.

**Table 3:** Participant characteristics: overall, characteristics were generally well balanced across the treatment groups

		Randomized to leukodepletion filter (*n* = 32)		Randomized to standard Filter (*n* = 31)		Overall (*n* = 63)	
		n	%	n	%	n	%
Age (years)	Median (IQR)	73.5	(69.6, 77.9)	72.0	(61.5, 76.9)	72.6	(66.1, 77.7)
Male		21/32	66%	18/31	58%	39/63	62%
Black ethnicity		2/32	6%	0/31	0%	2/63	3%
BMI	Median (IQR)	27.0	(24.4, 32.3)	29.7	(26.0, 34.6)	27.8	(25.5, 33.3)
EuroSCORE	Median (IQR)	6.0	(5.0, 7.0)	6.0	(3.0, 7.0)	6.0	(4.0, 7.0)
Cleveland CSA-AKI score	Median (IQR)	2.0	(1.0, 3.0)	2.0	(2.0, 3.0)	2.0	(1.0, 3.0)
ECHO/ANGIOGRAM REPORT							
LV function	Good (> 50%)	28/32	88%	26/31	84%	54/63	86%
	Moderate (30–50%)	4/32	13%	5/31	16%	9/63	14%
Extent of coronary disease	None/not investigated	22/32	69%	20/31	64%	42/63	66%
	Single	2/32	6%	5/31	16%	7/63	11%
	Double	3/32	9%	4/31	13%	7/63	11%
	Triple	5/32	16%	2/31	6%	7/63	11%
> 50% disease in left main stem		2/32	6%	2/31	6%	4/63	6%

The median age of participants was 72.6 years (interquartile range (IQR) 66.1 to 77.7) and 39/63 (62%) were male. The median additive European System for Cardiac Operative Risk Evaluation score (EuroSCORE) was 6.0 (IQR, 4.0 to 7.0) and the median Cleveland AKI severity score was 2.0 (IQR, 1.0 to 3.0). Overall, characteristics were well balanced across the treatment groups. The groups were also equivalent in terms of preoperative co morbidities, ventricular function and the extent of coronary artery disease based on pre-operative angiogram.

The operative data are demonstrated in Table 4. Isolated valve operations accounted for 33/62 (53%) procedures; the remainder included other procedures, mainly valve and coronary artery bypass graft (CABG) (21/62; 34%). The majority of patients had an aortic valve replacement (47/62; 76%). The peri-operative anesthetic management was standardized and bypass data was compatible. The median (IQR) total duration of CPB in minutes for the LG-6 group was 119.0 (97.0, 151.0) versus 124.0 (107.0, 141.0) for the standard filter group.

**Table 4:** Operative details: the bypass data were compatible

Operation detail		Randomized to leukodepletion filter (*n* = 32)		Randomized to standard filter (*n* = 31)		Overall (*n* = 63)	
		n	%	n	%	n	%
Type of surgery	Valve	15/31^a^	48%	18/31	58%	33/62	53%
	CABG and valve	11/31^a^	35%	10/31	32%	21/62	34%
	Valve and other	5/31^a^	16%	3/31	10%	8/62	13%
Valve replacement		26/31^a^	84%	25/31	81%	51/62	82%
Valve repair		8/31^a^	26%	7/31	23%	15/62	24%
Valve Replacement Details	Aortic	23/26	88%	24/25	96%	47/51	92%
	Mitral	3/26	12%	1/25	4%	4/51	8%
Valve Repair Details	Mitral	4/8	50%	5/7	71%	9/15	60%
	Tricuspid	3/8	38%	2/7	29%	5/15	33%
	Pulmonary	1/8	13%	0/7	0%	1/15	7%
BYPASS DATA							
Total CPB duration (minutes)	Median (IQR)	119.0	(97.0, 151.0)	124.0	(107.0, 141.0)	123.5	(99.0, 146.0)

Details on missing data (Leukodepletion, Standard): (1, 0)

*IQR* interquartile

^a^1 patient in the leukodepletion filter group was missing CPB specific data, as this patient was an open and shut case

Study outcomes are summarized in Table 5.

**Table 5:** Post-operative primary and secondary outcomes

		Randomized to leukodepletion filter (*n* = 32)		Randomized to standard filter (*n* = 31)		Overall (*n* = 63)		
		n	%	n	%	n	%	(95% CI)
**PRIMARY OUTCOME:**								
**Any AKI**	At any time	14/32	44%	7/31	23%	21/63	33%	(0.22, 0.46)
	Prior to discharge	14/29	48%	6/25	24%	20/54	37%	
	Between discharge and 6 weeks ~	0/26	0%	1/24	4%	1/50	2%	
Highest AKI stage	Stage 1	9/14	64%	4/6	67%	13/20	65%	
	Stage 2	4/14	29%	1/6	17%	5/20	25%	
	Stage 3	1/14	7%	1/6	17%	2/20	10%	
First instance of AKI	Day 1	9/14	64%	2/6	33%	11/20	55%	
	Day 2	4/14	29%	3/6	50%	7/20	35%	
	Day 3 to discharge	1/14	7%	1/6	17%	2/20	10%	
Maximum AKI stage	Day 1	6/14	43%	0/6	0%	6/20	30%	
	Day 2	7/14	50%	5/6	83%	12/20	60%	
	Day 3 to discharge	1/14	7%	1/6	17%	2/20	10%	
**SECONDARY OUTCOMES:**								
Need for haemodialysis		1/32	3%	0/31	0%	1/63	2%	(0.0,0.1)
In hospital mortality		2/32	6%	0/31	0%	2/63	3%	(0.0,0.1)
Infection complications		5/32	16%	4/31	13%	9/63	14%	(0.1,0.3)
Length of post-operative stay	Median (IQR) days	7.7	(5.8, 13.9)	8.8	(5.9, 11.7)	7.9	(5.9, 12.0)	
*CICU length of stay*	Median (IQR) days	47.3	(24.8, 100.0)	48.5	(26.6, 92.8)	48.5	(25.0, 98.3)	
*Ward length of stay*	Median (IQR) days	120.8	(94.0, 168.8)	141.5	(92.0, 188.4)	121.3	(93.5, 187.0)	

The incidence of post-operative AKI was reported in 21/63 (33%) participants (95% CI 22 to 46%). The incidence was higher in the leukodepletion filter group (14/32; 44% versus 7/31; 23%, risk difference 21, 95% CI − 2 to 44%).

Most patients developed stage 1 AKI (13/20; 65%), whereas only 10% were classified with stage 3 AKI. One patient in the leukodepletion filter group required haemodialysis. Diuretic use and fluid balance over the first 5 days were similar in the two groups. Only four patients were not given diuretics during this time.

There was no clear association between the incidence, severity of AKI and the secondary outcome of length of stay observed between the two groups (Table 5).

**Fig. 2:**
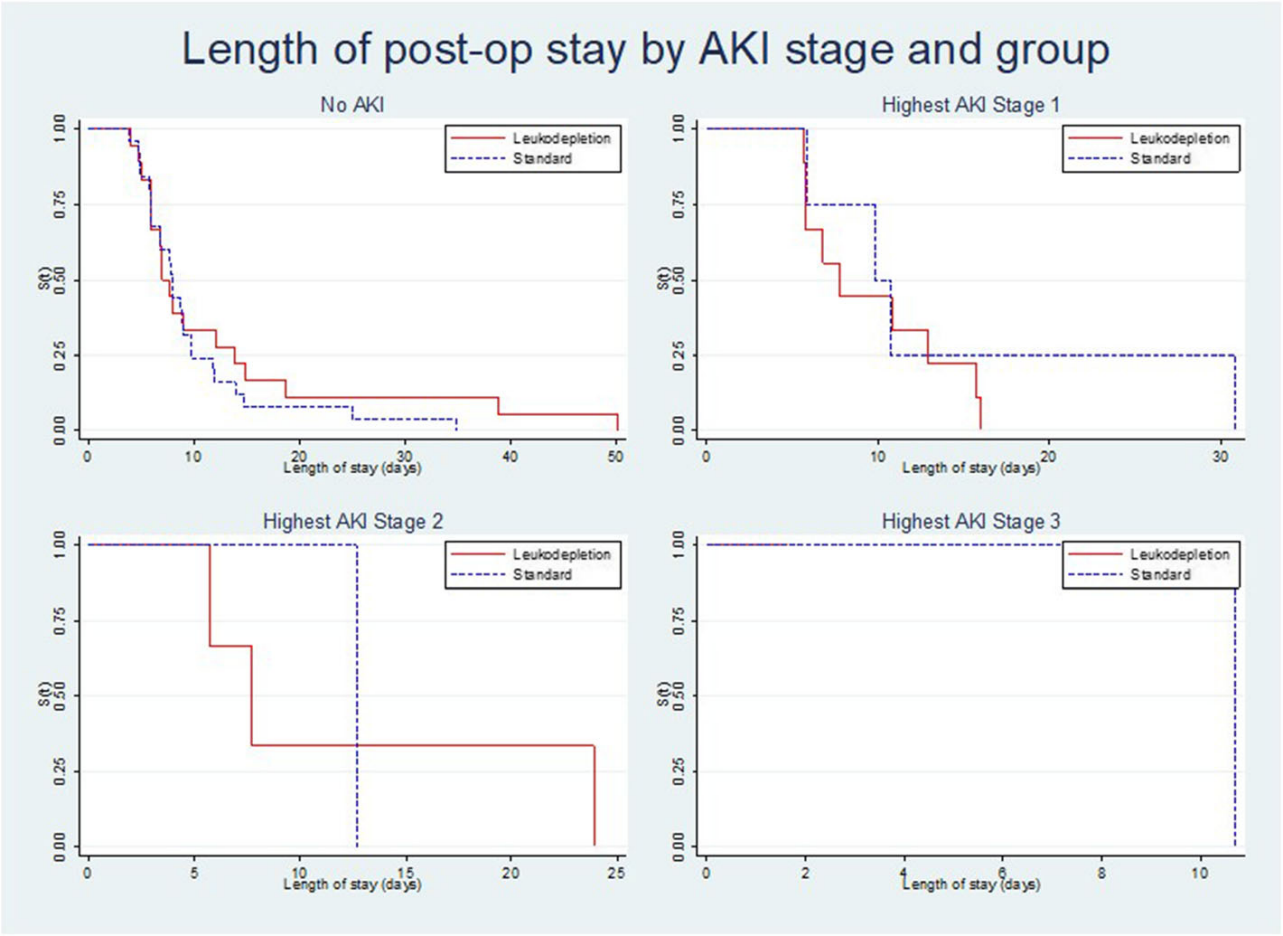
Probability of remaining in hospital by AKI stage for each of leukodepletion versus the standard filter group. Kaplan-Meier plot showing no clear association between the incidence, severity of AKI and the secondary outcome of length of stay observed between the two groups and highest AKI stage

Hospital stay by highest AKI stage is illustrated in Fig. 2. The median length of stay in intensive care was 47.3 h in the leukodepletion filter group and 48.5 h in the standard group. The median overall post-operative hospital stay was 7.7 days and 8.8 days respectively. Two patients in the leukodepletion filter group died during their post-operative stay. This was not directly related to the use of the filter. Infective complications occurred in 9/63 (14%) patients, with a similar incidence in both groups (leukodepletion filter 16%, standard filter 13%).

**Fig. 3:**
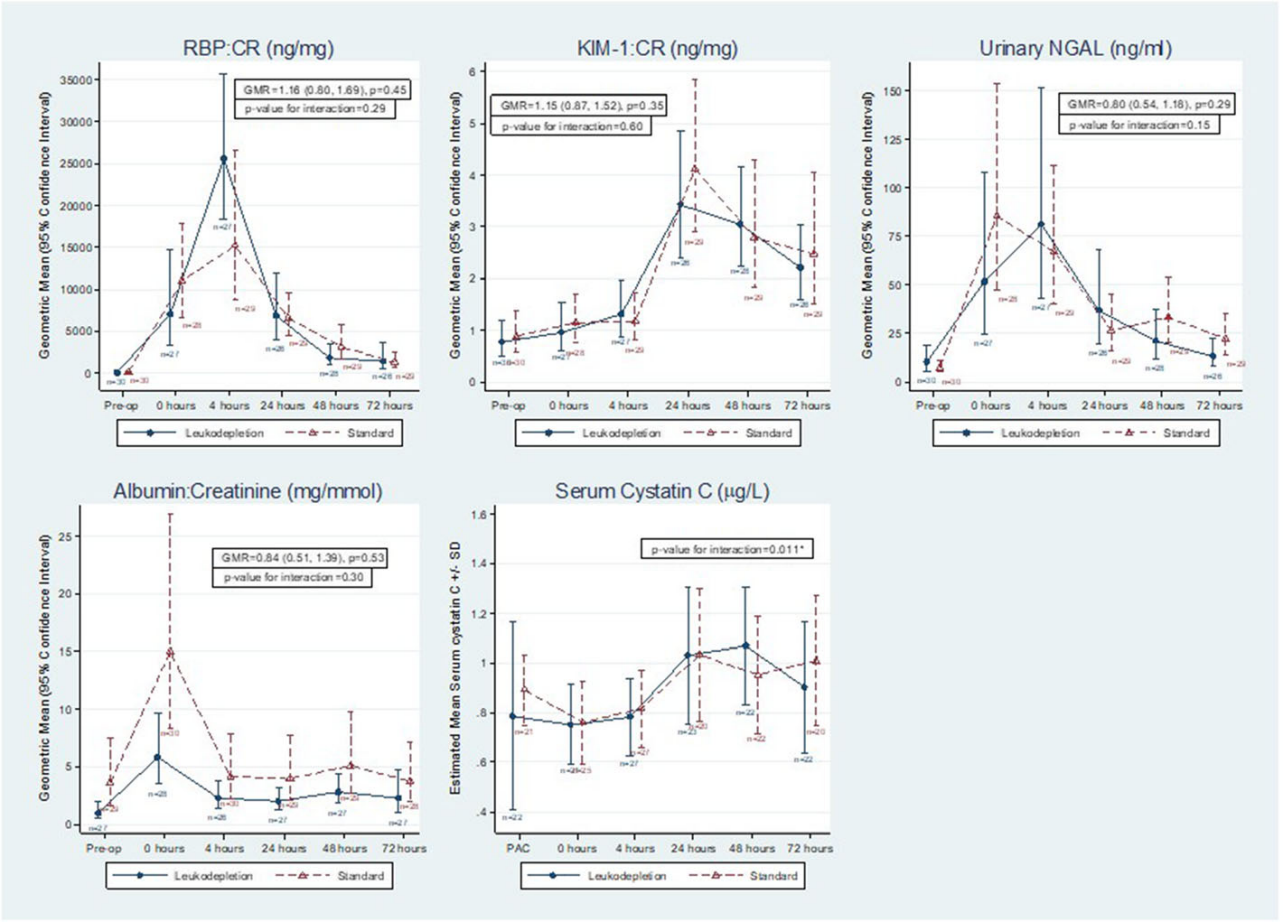
Relationship between the biomarkers of acute kidney injury studied in urine and serum with time. Serum Cystatin C was the only marker that showed significant raise in leukodepleated patients at 48 h post-surgery. (RBP = Retinol binding protein, KIM-1 = Kidney injury molecule − 1, Alb = micro Albumin, NGAL = Neutrophil gelatinase-associated lipocalin)

Biomarker comparisons are summarized in Fig. 3.

The urinary RBP:Cr and KIM-1:Cr ratios were, on average, tended to be higher in the leukodepletion filter group compared to standard filter group (geometric mean ratio (GMR) 1.16, 95% CI 0.80 to 1.69, *p* = 0.45 and 1.15, 95% CI 0.87 to 1.52, *p* = 0.35 respectively). A proportion of samples were below the limit of detection for KIM-1:Cr, i.e. below 0.156 ng/ml (46/338 observations, 13.7%). Urinary NGAL and Alb:Cr ratio were, on average, lower in the leukodepletion group (geometric mean ratio (GMR) 0.80, 95% CI 0.54–1.18, *p* = 0.29 and 0.84, 95% CI 0.51–1.39, *p* = 0.53). None of the differences were statistically significant. Serum Cystatin C concentration was the only biomarker where the difference between the groups varied significantly with time (*p* = 0.011). After adjustment for pre-operative AKI severity, the data suggest a possible difference at 48 h (mean difference 0.11, 95% CI 0.00 to 0.23, *p* = 0.068) with higher average values in the leukodepletion groups, but not at other times.

Comprehensive resource use data were collected. EQ-5D- 5 L scores were missing for ≤3 (5%) patients at each time point (patients who died were assigned a score of zero for each time point after death). The mean (SD) EQ-5D single summary index scores for the cohort as a whole were 0.82 (0.15) preoperatively, and 0.72 (0.24), 0.78 (0.22) and 0.85 (0.22) at 5 days, 6 weeks and 12 weeks respectively, indicating that HRQoL improved with time after surgery. The MLHF scores showed a similar pattern.

The overall scores and the scores for both the physical and emotional subscales reduced from 5 days to 12 weeks indicating HRQoL improved over time (mean (SD)): overall score at baseline 37.4 (23.0), 5 days 45.6 (25.1), 6 weeks 29.6 (19.6), 12 weeks 16.2 (16.3). Resource use during follow up was captured for 36/38 (95%) participants at 6 weeks and 57/61 (93%) participants at 3 months.

Overall, 3% of resource use data were missing: 1% for the in-patient period (which had the greatest number of resource use variables), 5% for follow up to 6 weeks, and 8% of follow up data to 3 months. On average, health related quality of life returned to pre-operative levels by 3 months.

A total of 203 adverse events (Supplementary material) were reported in 58 participants in the first 3 months after surgery, 66 of which were classified as serious adverse events (SAE). Forty SAEs were reported in 13 patients in the leucodepletion filter group compared to 26 SAEs in 13 patients in the standard filter group. Of the 203 adverse events reported, 182 were expected complications after cardiac surgery, i.e. they were listed in the study protocol, and 21 were unexpected. Approximately one third of SAEs occurred after hospital discharge; SVT/AF requiring treatment was the most frequently reported SAE, which occurred in 5 participants (8%). These differences however did not reach significance.

## Discussion

Cochrane systematic review by the authors, failed to identify good quality trials in support of using leukodepletion in cardiac surgery in order to inform recommendations for a change in practice for its routine use [18]. Therefore this randomized trial was intended as a feasibility experiment to estimate key clinical, sub clinical and statistical parameters needed to design a larger more definitive trial in this subject.

However the results did not support the hypothesis that leukodepletion throughout cardiopulmonary bypass would reduce inflammatory response by removing activated leukocytes.

The incidence of the primary outcome of AKI was almost 1.5 times higher in the leukodepletion group compared to the standard. Serum levels of secondary outcome of Cystatin C showed evidence of subclinical AKI in the leukodepletion group as compared to the standard. However this did not translate into a difference in hospital stay or post procedural quality of life between the groups.

The study was conducted in a single centre so it may be more challenging to achieve similar compliance in a multi-centre setting. However, as was demonstrated in the Titre-2 trial, with good engagement with study centers and participants excellent compliance with follow-up is achievable [30].

The incidence of AKI (the primary outcome) was 33% which is consistent with previous reports [7], but notably higher than the 13.2% incidence reported in the Titre-2 trial which recruited 2000 cardiac surgery patients across 17 UK centers [31]. This could be explained by the differences in study cohort (41% of patients in Titre-2 underwent isolated CABG surgery). In the subgroup of the Titre-2 cohort who underwent valve surgery (603 participants) the incidence was 13.8 and 4.3% were AKI stage 1 (personal communication).

In ROLO the primary outcome could not be calculated definitively for 9/63 participants due to the Creatinine not being measured at the 6 week follow-up visit. This is a limitation, but with only one patient (2%) developing AKI after discharge, not apparent immediately after surgery, it is unlikely that the cases with missing data would have experienced AKI.

Blinding of clinical teams to the intervention was successful and the treatment was delivered as planned, with only one cross-over for clinical reasons. There were 4 withdrawals after consent due to surgeon preference, reflecting the challenge of conducting a clinical trial in surgery.

Serum Cystatin C concentration was the only biomarker where the difference between the groups varied significantly with time indicative of renal insult at 48 h. The anticipated 1.5 fold reduction in RBP:Cr in the leukodepletion group [15] was not borne out in this study. Contrary to expectation the biomarker concentration was on average 16% higher in the leucodepletion filter group. The Alb:Cr ratio was lower in the leucodepletion group, but on average less than anticipated (GMR 0.84 against a target of 0.66). A significant proportion of KIM-1, samples were below the detection limit i.e. below 0.156 ng/ml (46/338 observations, 13.7%). However a sensitivity analysis omitting values below the limit of detection was consistent with the primary analysis.

Numerous factors contribute to the development of renal injury, including reductions in renal blood flow, actions of nephrotoxic drugs, injury to the proximal tubule epithelial cells, pro-inflammatory responses of renal endothelial cells, influx and activation of inflammatory leukocytes that further reduces renal blood flow through vascular congestion and promotes kidney injury [32, 33]. Although numerous studies have demonstrated the detrimental role of neutrophils, recent reports have uncovered a protective and possibly therapeutic role of neutrophils in AKI. Studies in animal models have revealed that neutrophils promote renal injury. In contrast, neutrophils are critical mediators of innate immunity. They respond rapidly (within minutes) to invading pathogens and to sites of tissue damage. Neutrophils clear invading pathogens by phagocytosis or by releasing toxic granules containing proteases and other enzymes and reactive oxygen species (ROS). For this reason, neutrophil depletion can lead to damage of host cells in the inflamed tissue. In several animal models, neutrophil accumulation in the injured kidney is a consistent and early finding and depletion of neutrophils or prevention of neutrophil tracking to the kidney reduces kidney injury. However, these results may be related to differences in experimental models and limitations to methods for neutrophil depletion. In addition neutrophils releasing pro-inflammatory cytokines, interferon IFN-γ and interleukin IL-17, and the chemokine CXCL1, in the injured kidney [34–36] and direct or indirect release of anti-inflammatory cytokines IL- 6 IL-10, etc. may suggest a more complex balance of pro and anti-inflammatory mechanism of neutrophils related pathogenesis of kidney injury.

## Conclusion

The ROLO trial has demonstrated that a higher incidence of AKI and raised biomarkers of renal injury are associated with the use of a leukodepletion filter. Addition of a leukodepletion filter to the bypass circuit for valvular heart surgery cannot be supported for the purpose of limiting post-operative renal dysfunction. Other interventions to reduce the incidence of this important complication should be explored.

## Supplementary Information

**Additional file 1.**

## Data Availability

The trial was run by Bristol clinical trials and evaluation unit through whom all supporting data could be made available.

## Abbreviations

AE: Adverse event - any undesirable event in a subject receiving treatment according to the protocol including occurrences which are not necessarily caused by or related to administration of the research procedures
AF: Atrial Fibrillation
AKI: Acute kidney injury - an acute increase in serum creatinine > 26.4 μmol/l or a percentage increase in serum creatinine of more than or equal to 50%
AR: Adverse reaction – any undesirable experience that has happened a subject while taking a drug that is suspected to be caused by the drug or drugs
ARDS: Acute respiratory distress syndrome
ARF: Acute renal failure
AVR: Aortic valve replacement/repair
BHI: Bristol Heart Institute
BRI: Bristol Royal Infirmary
BRU: Biomedical Research Unit
CABG: Coronary artery bypass grafting
CAD: Coronary artery disease
CKD Stage: International classification of chronic kidney disease
CPB: Cardiopulmonary bypass - a technique that temporarily takes over the function of the heart and lungs during surgery, maintaining the circulation of blood and the oxygen content of the body whilst allowing surgeons to access static cardiac tissue
CSA-AKI: Cardiac surgery associated acute kidney injury
CTEU: Clinical Trials and Evaluation Unit
CVVH: Continuous veno-venous hemofiltration
DM: Diabetes mellitus
DMSC: Data monitoring and safety committee
ECG: Graphical representation of electrical activity of the heart over time, as recorded by an electrocardiograph
eGFR: Estimated glomerular filtration rate: derived from gender, age, ethnicity and serum creatinine
ELISA: Enzyme-linked immunosorbent assay
ESRF: End stage renal disease requiring dialysis, CKD stage 5
HDU HRQoL: High dependency unit, Health-related quality of life
ICH-GCP: International conference for harmonisation of good clinical practice
ICU: Intensive care unit
IL-: Interleukin- a validated specific marker of acute kidney injury
ITT: Intension to treat
KDIGO: Kidney Disease Improving Global Outcomes
KIM-1: Kidney Injury Molecule-1
LDH: Lactate dehydrogenase – an enzyme often used as a marker of tissue breakdown; high levels of LDH indicate tissue damage
LFT: Liver function test
MACE: Major cardiac adverse event
MI: Myocardial infarction
MLHF: Minnesota living with heart failure
MRC: Medical Research Council
NCEPOD: National Confidential Enquiry into Patient Outcome and Death
NGAL: Neutrophil gelatinase associated lipocalin – a specific marker of acute kidney injury
NIHR: National Institute for Health Research
RCT: Randomised controlled trial
RIFLE: Risk, Injury, Failure, Loss of kidney function
REC: Research ethics committee
SAE: Serious adverse event - events which result in death, are life threatening, require hospitalisation or prolongation of hospitalisation, result in persistent or significant disability or incapacity
SAR: Serious adverse reaction
SIRS: ^Sy^stemic inflammatory response syndrome
SOP: Standard operating procedure
SSAR: Suspected serious adverse reaction
SUSAR: Suspected unexpected serious adverse reaction - an untoward medical occurrence suspected to be related to a medicinal product that is not consistent with the applicable product information and is serious
SVT: Supra ventricular tachycardia
TIA: Transient ischemic attack
TMG: Trial management group
TSC: Trial steering committee
UH Bristol: University Hospitals Bristol NHS Foundation Trust
