# Supplementary material for: Is there a renoprotective value to leukodepletion during heart valve surgery? A randomized controlled trial (ROLO)
Source: J Cardiothorac Surg. 2021 Mar 26;16:58. doi: 10.1186/s13019-021-01402-4 (PMC8004389; doi:10.1186/s13019-021-01402-4)
Supplement: Supplementary file 1 — Additional file 1. [file 13019_2021_1402_MOESM1_ESM.docx]

**Supplementary Material:** Complications and serious complications within index hospital admission**.**

|  | Randomized to leukodepletion filter (n=32) | | | Randomized to standard filter  (n=31) | | | | | Overall  (n=63) | | | |
| --- | --- | --- | --- | --- | --- | --- | --- | --- | --- | --- | --- | --- |
|  | Complications | | Serious complications | Complications | | | Serious complications | | Complications | | Serious complications | |
|  | Events/ Patients | % | Events/  Patients | % | Events/ Patients | % | Events/ Patients | % | Events/ Patients | % | Events/ Patients | % |
| Any event | 100/30 | 94% | 30/8 | 25% | 78/27 | 87% | 11/9 | 29% | 178/57 | 90% | 41/17 | 27% |
| Any “Expected” Event | 90/30 | 94% | 20/6 | 19% | 74/27 | 87% | 7/6 | 19% | 164/57 | 90% | 27/12 | 19% |
| Peri-operative MI | 1/1 | 3% | 1/1 | 3% | 0/0 | 0% | 0/0 | 0% | 1/1 | 2% | 1/1 | 2% |
| Cardiac arrest | 2/2 | 6% | 2/2 | 6% | 1/1 | 3% | 1/1 | 3% | 3/3 | 5% | 3/3 | 5% |
| Any arrhythmias |  |  |  |  |  |  |  |  |  |  |  |  |
| SVT/AF (requiring treatment)* | 15/15 | 48% | 1/1 | 3% | 13/12 | 39% | 0/0 | 0% | 28/27 | 44% | 1/1 | 2% |
| VF/VT (requiring treatment)* | 1/1 | 3% | 1/1 | 3% | 0/0 | 0% | 0/0 | 0% | 1/1 | 2% | 1/1 | 2% |
| Pacing | 20/20 | 63% | 3/3 | 9% | 18/18 | 58% | 1/1 | 3% | 38/38 | 60% | 4/4 | 6% |
| Any hemodynamic support (post-operative) |  |  |  |  |  |  |  |  |  |  |  |  |
| Inotropes | 23/23 | 72% | 1/1 | 3% | 23/23 | 74% | 0/0 | 0% | 46/46 | 73% | 1/1 | 2% |
| IABP | 2/2 | 6% | 1/1 | 3% | 0/0 | 0% | 0/0 | 0% | 2/2 | 3% | 1/1 | 2% |
| Pulmonary artery catheter | 5/5 | 16% | 1/1 | 3% | 2/2 | 6% | 0/0 | 0% | 7/7 | 11% | 1/1 | 2% |
| Vasodilator | 2/2 | 6% | 1/1 | 3% | 0/0 | 0% | 0/0 | 0% | 2/2 | 3% | 1/1 | 2% |
| Low cardiac output | 2/2 | 6% | 1/1 | 3% | 1/1 | 3% | 0/0 | 0% | 3/3 | 5% | 1/1 | 2% |
| Any pulmonary complications |  |  |  |  |  |  |  |  |  |  |  |  |
| Re-intubation† | 1/1 | 3% | 1/1 | 3% | 1/1 | 3% | 1/1 | 3% | 2/2 | 3% | 2/2 | 3% |
| Tracheotomy^ | 1/1 | 3% | 1/1 | 3% | 2/1 | 3% | 0/0 | 0% | 3/2 | 3% | 1/1 | 2% |
| Mask CPAP^ | 5/3 | 10% | 2/1 | 3% | 0/0 | 0% | 0/0 | 0% | 5/3 | 5% | 2/1 | 2% |
| Pneumothorax or effusion requiring drainage^ | 1/1 | 3% | 0/0 | 0% | 6/6 | 19% | 0/0 | 0% | 7/7 | 11% | 0/0 | 0% |
| Any GI complications |  |  |  |  |  |  |  |  |  |  |  |  |
| Other GI e.g. laparotomy, obstruction^ | 1/1 | 3% | 0/0 | 0% | 0/0 | 0% | 0/0 | 0% | 1/1 | 2% | 0/0 | 0% |
| Any neurological complications |  |  |  |  |  |  |  |  |  |  |  |  |
| Permanent stroke^ | 0/0 | 0% | 0/0 | 0% | 1/1 | 3% | 1/1 | 3% | 1/1 | 2% | 1/1 | 2% |
| Any thromboembolic complications |  |  |  |  |  |  |  |  |  |  |  |  |
| Any wound dehiscence requiring rewiring or treatment ^ | 0/0 | 0% | 0/0 | 0% | 1/1 | 3% | 0/0 | 0% | 1/1 | 2% | 0/0 | 0% |
| Infective complications (confirmed by SIRS) | 5/5 | 16% | 0/0 | 0% | 4/4 | 13% | 2/1 | 3% | 9/9 | 14% | 2/1 | 2% |
| Respiratory^ | 3/3 | 10% | 0/0 | 0% | 7/7 | 23% | 0/0 | 0% | 10/10 | 16% | 0/0 | 0% |
| Wound^ | 0/0 | 0% | 0/0 | 0% | 1/1 | 3% | 0/0 | 0% | 1/1 | 2% | 0/0 | 0% |
| Renal Complications* | 1/1 | 3% | 1/1 | 3% | 0/0 | 0% | 0/0 | 0% | 1/1 | 2% | 1/1 | 2% |
| Bleeding requiring reoperation* | 2/2 | 6% | 2/2 | 6% | 1/1 | 3% | 1/1 | 3% | 3/3 | 5% | 3/3 | 5% |
| Any “Unexpected” Event | 10/7 | 22% | 10/7 | 22% | 4/3 | 10% | 4/3 | 10% | 14/10 | 16% | 14/10 | 16% |
| Confusional state | 1/1 | 3% | 1/1 | 3% | 1/1 | 3% | 1/1 | 3% | 2/2 | 3% | 2/2 | 3% |
| Death | 2/2 | 6% | 2/2 | 6% | 0/0 | 0% | 0/0 | 0% | 2/2 | 3% | 2/2 | 3% |
| Dyspnoea | 1/1 | 3% | 1/1 | 3% | 0/0 | 0% | 0/0 | 0% | 1/1 | 2% | 1/1 | 2% |
| Generalized tonic-clonic seizures | 4/1 | 3% | 4/1 | 3% | 0/0 | 0% | 0/0 | 0% | 4/1 | 2% | 4/1 | 2% |
| Incontinent | 0/0 | 0% | 0/0 | 0% | 1/1 | 3% | 1/1 | 3% | 1/1 | 2% | 1/1 | 2% |
| Loss of consciousness | 0/0 | 0% | 0/0 | 0% | 1/1 | 3% | 1/1 | 3% | 1/1 | 2% | 1/1 | 2% |
| Retention of urine | 1/1 | 3% | 1/1 | 3% | 1/1 | 3% | 1/1 | 3% | 2/2 | 3% | 2/2 | 3% |
| Thrombocytopenia | 1/1 | 3% | 1/1 | 3% | 0/0 | 0% | 0/0 | 0% | 1/1 | 2% | 1/1 | 2% |
